# Ultrasensitive Biosensors Using Enhanced Fano Resonances in Capped Gold Nanoslit Arrays

**DOI:** 10.1038/srep08547

**Published:** 2015-02-24

**Authors:** Kuang-Li Lee, Jhih-Bin Huang, Jhih-Wei Chang, Shu-Han Wu, Pei-Kuen Wei

**Affiliations:** 1 Research Center for Applied Sciences, Academia Sinica, Taipei 11529, Taiwan; 2 Institute of Physics, Academia Sinica, Taipei 11529, Taiwan; 3 Department of Mechanical and Mechatronic Engineering, National Taiwan Ocean University, Keelung 20224, Taiwan; 4 Institute of Biophotonics, National Yang-Ming University, Taipei 11221, Taiwan; 5 Department of Optoelectronics, National Taiwan Ocean University, Keelung 20224, Taiwan

## Abstract

Nanostructure-based sensors are capable of sensitive and label-free detection for biomedical applications. However, plasmonic sensors capable of highly sensitive detection with high-throughput and low-cost fabrication techniques are desirable. We show that capped gold nanoslit arrays made by thermal-embossing nanoimprint method on a polymer film can produce extremely sharp asymmetric resonances for a transverse magnetic-polarized wave. An ultrasmall linewidth is formed due to the enhanced Fano coupling between the cavity resonance mode in nanoslits and surface plasmon resonance mode on periodic metallic surface. With an optimal slit length and width, the full width at half-maximum bandwidth of the Fano mode is only 3.68 nm. The wavelength sensitivity is 926 nm/RIU for 60-nm-width and 1,000-nm-period nanoslits. The figure of merit is up to 252. The obtained value is higher than the theoretically estimated upper limits of the prism-coupling SPR sensors and the previously reported record high figure-of-merit in array sensors. In addition, the structure has an ultrahigh intensity sensitivity up to 48,117%/RIU.

Surface plasmon resonance (SPR) sensing is a real-time and label-free detection technique which has been employed in applications including medical diagnostics, environmental monitoring, and food safety^1^,^2^,^3^,^4^. The most common method to induce SPR on the gold surface is to utilize an optical prism, known as the Kretschmann configuration. On the basis of this SPR excitation technique, commercial instruments enable real-time and label-free measurements of biomolecular binding affinity. In addition to the prism coupling method, metallic nanostructures offer a simple way for SPR excitation^5^,^6^,^7^,^8^,^9^. Recently, periodic gold nanohole arrays or nanoslit arrays have been utilized for biosensing applications^10^,^11^,^12^,^13^,^14^,^15^. Compared to prism-based SPR sensors, gold nanostructures benefit from having a small detection volume and normal light incidence. They provide a feasible way to achieve chip-based, high-throughput and label-free detection for modern DNA and protein microarrays^16^,^17^.

Plasmonic sensors capable of highly sensitive and multiplexed detection with a mass-fabrication process and low fabrication cost are desirable to many applications. In order to reach the best sensing performance, sensors having ultrahigh quality factors are required. A narrower resonance linewidth allows a lower molecular concentration to be detected. The linewidth is related to the surface plasmon propagation loss. Recently, a mass-fabrication process for making high-quality metallic nanostructures was proposed by using a thermal-annealing nanoimprint method^18^,^19^,^20^,^21^. The thermal-annealing nanostructures have smoother metal surfaces and larger gold grains^18^,^22^ which reduce surface plasmon propagation loss and result in a sharp linewidth. Another approach to achieve sharp spectral response is based on Fano resonances^23^,^24^,^25^. The Fano resonance exhibits a distinctly asymmetric shape which arises from the spectral overlapping between a broad resonance and a narrow discrete resonance^24^. The Fano resonances have been extensively studied in nanoparticles^9^, plasmonic nanostructures^25^,^26^,^27^,^28^,^29^,^30^,^31^ and metamaterial systems^32^.

Here, we utilized the thermal-embossing template-stripping method to fabricate large-area capped gold nanoslits on polymer films with low cost and ultrahigh SPR sensitivities. We found that a transverse magnetic (TM)-polarized wave in the capped nanoslits generated extremely sharp and asymmetric Fano resonances in transmission spectra. The full width at half-maximum bandwidth (FWHM) was only 3.68 nm (0.0026 eV) and the wavelength sensitivity was 926 nm/RIU (0.653 eV/RIU) for 1,000-nm-period nanoslits. In addition, the extremely sharp resonance leads to an ultrahigh intensity sensitivity up to 48,117%/RIU. Compared to previous single-layer SPR sensors, the proposed structure has a much narrower bandwidth. It reaches a figure of merit (FOM) up to 252 (FOM_E_ = 251). We attribute such high sensitivity to enhanced resonant effects by the gold capping layer. It caps the gap plasmon in nanoslits, resulting in an enhanced cavity mode. In addition, the outside surface plasmon wave between nanoslits has a better confinement, resulting in an enhanced SPR mode. The strength of the Fano coupling between these two modes can be tuned by the thickness of the gold film and slit width. An optimal Fano coupling is achieved when the capping layer is close to the height of the nanoslits.

## Results

### Optical properties of the capped gold nanoslits

**Figure 1:**
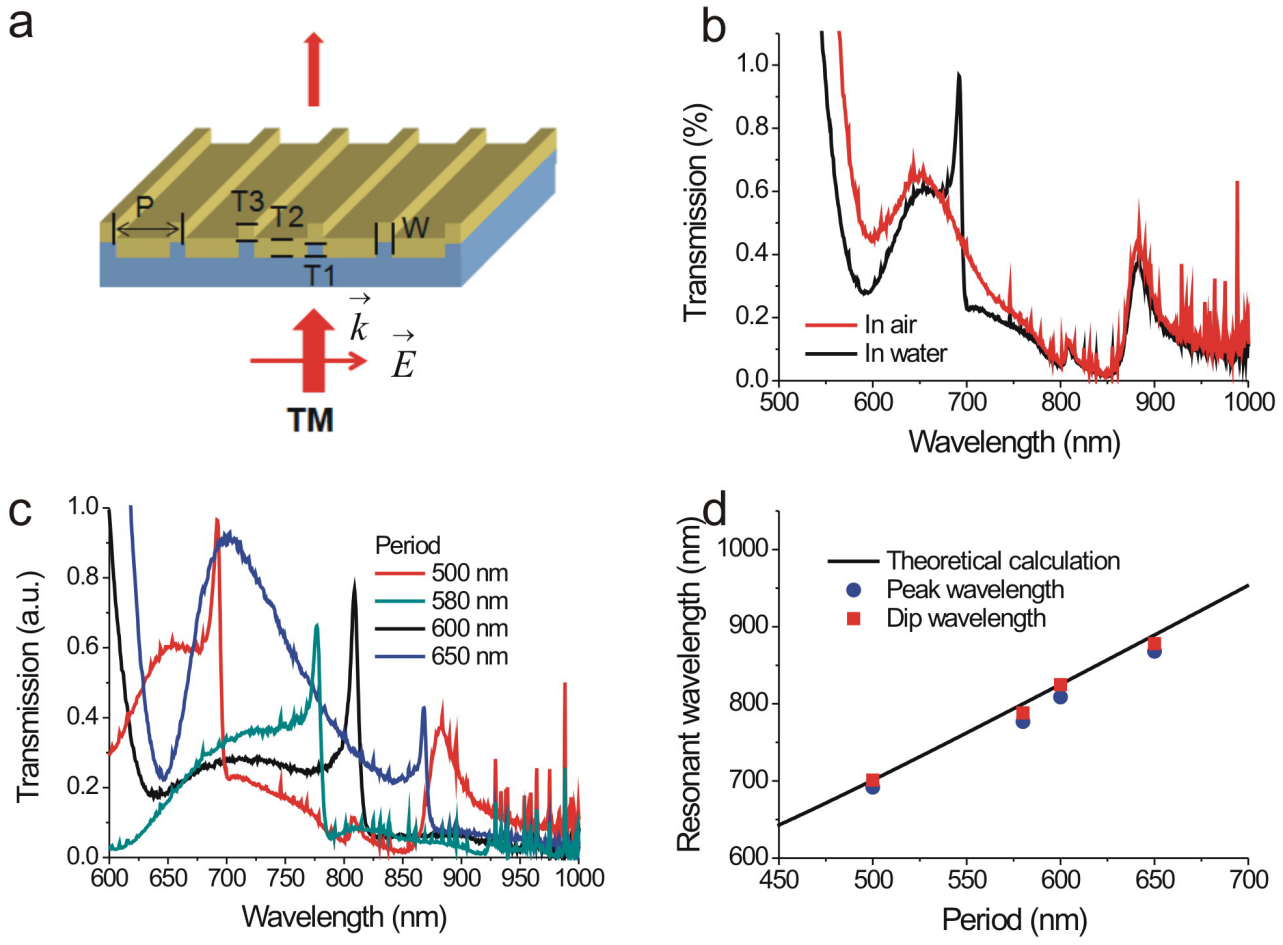
Optical properties of the capped gold nanoslits. (a) A schematic configuration, depicting the geometrical parameters of the capped gold nanoslits, as well as the direction of the TM-polarized incident light with E and k vectors. (b) The measured transmission spectra of the 500-nm-period capped gold nanoslits in air and water for normally-incident TM-polarized light. We chose T1 = 55 nm, T2 = T3 = 80 nm, W = 60 nm, and P = 500 nm for the structure. (c) The transmission spectra of the capped nanoslits with various periods (from 500 to 650 nm) in water for a TM-polarized wave. (d) Experimental wavelengths of Fano resonances and theoretical (equation (1)) resonance wavelengths of BW-SPPs as a function of the period.

Figure 1a shows the schematic configuration, depicting the geometrical parameters of the capped gold nanoslits, as well as the direction of the TM-polarized incident light with E and k vectors. Figure 1b shows the measured transmission spectra of the 500-nm-period capped gold nanoslits in air and water for normally incident TM-polarized light. We chose T1 = 55 nm, T2 = T3 = 80 nm, W = 60 nm, and P = 500 nm for the structure. There are transmission peaks and dips in the spectrum due to the couplings of cavity resonances in nanoslits and BW-SPPs on the periodic metal surface. The cavity mode has a broadband resonance in the transmission spectrum. The resonant condition can be estimated by the Fabry-Perot resonance condition^33^. It is affected by the aperture size and cavity depth. On the other hand, the BW-SPP occurs on the outside surface when the Bragg condition is satisfied^34^. For a normally incident light, the condition for a 1-D array is dominated by the period and can be described by,
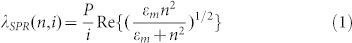
where i is the resonant order, P is the period of the nanostructure, ε_m_ is the dielectric constant of the metal and n is the environmental refractive index. The interaction between cavity resonances in nanoslits and BW-SPPs creates a Fano-like resonance profile consisting of a minimum, close to the position predicted by equation (1), and an adjacent maximum. The resonant wavelength of the BW-SPPs at the PC/gold interface (ε_m_ = −29 + 2.0i for gold at 800 nm, i = 1, n = 1.584 and P = 500 nm) is 832 nm. The theoretical resonant wavelength of the BW-SPP for 500-nm-period array is 550 nm at the air/gold interface (ε_m_ = −5.8 + 2.1i for gold at 549 nm, n = 1 and P = 500 nm). The expected resonance at the air/gold interface is not found due to the large propagation loss of surface plasmon at this wavelength^21^. When the array was covered with water, there were two Fano resonances in the water spectrum. The resonant dip of Fano resonance at the water/gold interface was at a wavelength of 692 nm. The resonant dip of the Fano resonance at the PC/gold interface remained unchanged. From equation (1), the resonant wavelength of the BW-SPPs at the water/gold interface is 704 nm (ε_m_ = −16.8 + 1.1i for gold at 705 nm, i = 1, n = 1.3320 and P = 500 nm). It is close to the experimental wavelength. Figure 1c shows the transmission spectra of the capped gold nanoslits with various periods in water for a TM-polarized wave. The couplings of cavity resonances in nanoslits and BW-SPPs create sharp Fano resonances at the water/gold interface. Obviously, the experimental wavelengths were close to the theoretical values as shown in Figure 1d.

### The mechanism and measurement/calculations of Fano resonances in capped gold nanoslit arrays

**Figure 2:**
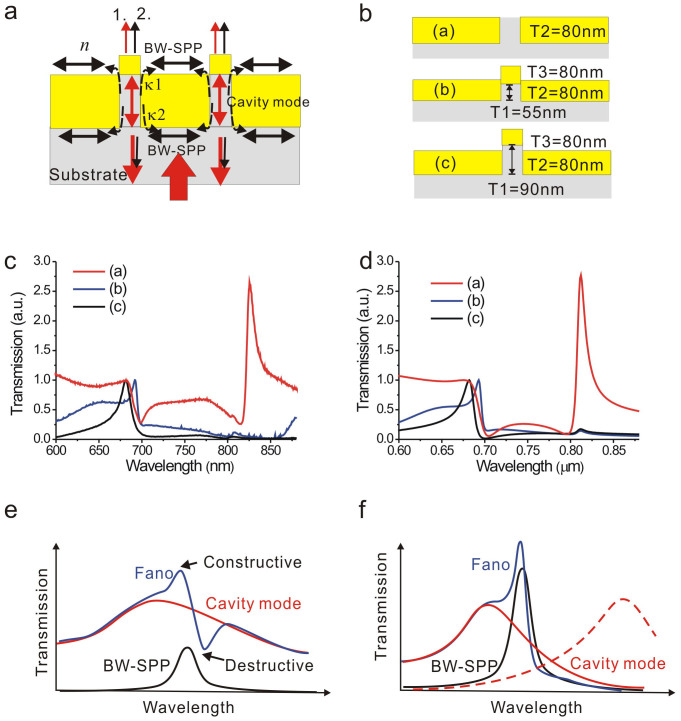
The mechanism and measurement/calculations of Fano resonances in capped gold nanoslit arrays. (a) The model of the Fano resonance resulted from the interference of the scattering cavity mode (1) and BW-SPP mode (2). The BW-SPP is a dark mode and the cavity mode is bright mode. There are two dark modes in the system. (b) A schematic configuration, depicting the geometrical parameters of the nanoslits, long-cavity capped nanoslits and short-cavity capped nanoslits. (c) The measured normalized transmission spectra of 500-nm-period gold nanostructures with different structure parameters in water for normally-incident TM-polarized light. (d) The calculated Fano resonance spectra for these three nanostructures using equation (2). The fitting parameters [λ_b_, γ_b_, λ_1_, γ_1_, q_1_, q_2_] are [0.72 μm, 0.2 μm^−1^, 0.695 μm, 0.025 μm^−1^, 0.5, 2.2] for non-capped nanoslits, [0.65 μm, 0.15 μm^−1^, 0.695 μm, 0.008 μm^−1^, 1, 0.25] for capped nanoslits (T1 = 55 nm) and [0.9 μm, 0.15 μm^−1^, 0.685 μm, 0.015 μm^−1^, 2, 0.25] for capped nanoslits (T1 = 90 nm). (e) A schematic illustration demonstrates Fano resonances in classic periodic gold nanoslits. (f) A schematic illustration demonstrates Fano resonances in capped gold nanoslits. The short-cavity mode has a large overlapping with the BW-SPP mode. A sharp Fano resonance profile is formed. On the other hand, the long-cavity mode is redshifted (dashed). There is a low interference effect in the structure.

Figure 2a shows the model for the capped gold nanoslit array. The incident light excites the cavity resonance (bright mode) in the nanoslit. The bright mode is coupled to the BW-SPP wave (dark mode) from the edges of top and bottom interfaces. The uncoupled light transmits through the nanoslits and capping layer, leading to a broadband transmission with the cavity spectrum (ray 1). On the other hand, the BW-SPP wave is scattered by the periodic grooves, leading to a narrowband transmission with the SPR spectrum (ray2). Both ray 1 and ray 2 form constructive and destructive interference, which results in an asymmetrical resonant spectrum. Such Fano resonant profile can be described by the following equation^35^:
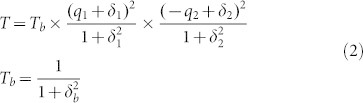
where T_b_ is the broadband resonance with a normalized wavelength, 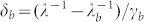. λ_b _is the cavity resonance wavelength and γ_b_ is the damping factor. q_1_ and q_2_ are Fano shape factors describing the interference between the BW-SPP and cavity scattering pathways. δ_1_ and δ_2_ are the normalized frequencies at the top and bottom surfaces, respectively. The corresponding resonant wavelength of the BW-SPP mode is λ_1_ with a damping factor, γ_1_ for the top surface (air or water). The resonant wavelength of the BW-SPP mode for the bottom surface (substrate) is λ_2_ with a damping factor, γ_2_. Figure 2b shows three cases of the gold nanoslit array. The first one is a classic nanoslit array. The second one is a capped nanoslit array with a short cavity length. The third is also a capped nanoslit with a long cavity length. Figure 2c shows the measured spectra of these three structures, single-layer nanoslit array and capped nanoslit arrays fabricated using different slit depths in Si molds. The gold thickness (80 nm), periodicity (500 nm), and slit width (60 nm) of nanoslit arrays are the same for all these structures. The different slit depths in the Si mold resulted in 90 and 55 nm cavity lengths, respectively. Figure 2d shows the calculated Fano resonance spectra. The fitting parameters for the bottom BW-SPP mode are the same for three cases, where λ_2_ = 0.81 μm and γ_2_ = 0.008 μm^−1^. The other parameters are indicated in Figures 2e and 2f. Figure 2e illustrates the interference effect in the Fano resonance spectrum. It can be seen that the classic nanoslit array has a low asymmetrical factor (q_1_ = 0.2) and larger damping factors (γ_b_, γ_1_) for both the cavity mode and BW-SPP mode on the top surface. Figure 2f illustrates the capped nanoslit array. This nanostructure has a higher asymmetrical factor (q_1_ = 1.1) and lower damping factors for both the cavity mode and BW-SPP mode. From the fitting results, the capping gold layer takes several advantages of Fano resonances. First, it improves the cavity mode resonance due to a higher reflection at the top interface. The resonant quality of BW-SPPs on the periodic metal surface is also increased. Second, the asymmetrical factor is increased because the longitudinal cavity mode is efficiently coupled to lateral BW-SPP mode. It is noted that the Fano resonance occurs when there is a spectral overlapping between the broadband and narrowband modes. For the long cavity mode (T1 = 90 nm), the cavity resonance is redshifted as indicated in the dashed line in Figure 2f. The Fano resonance is not efficient. The transmission of the long-cavity capped nanoslit array is dominant by the BW-SPP mode. When the slit thickness is reduced to 55 nm, the cavity mode and BW-SPP mode has similar resonant peaks. The strong coupling of the cavity resonance and BW-SPPs forms an extremely sharp and asymmetric Fano resonance.

### The effect of the slit width on the transmission spectra of long- and short-cavity capped nanoslits

**Figure 3:**
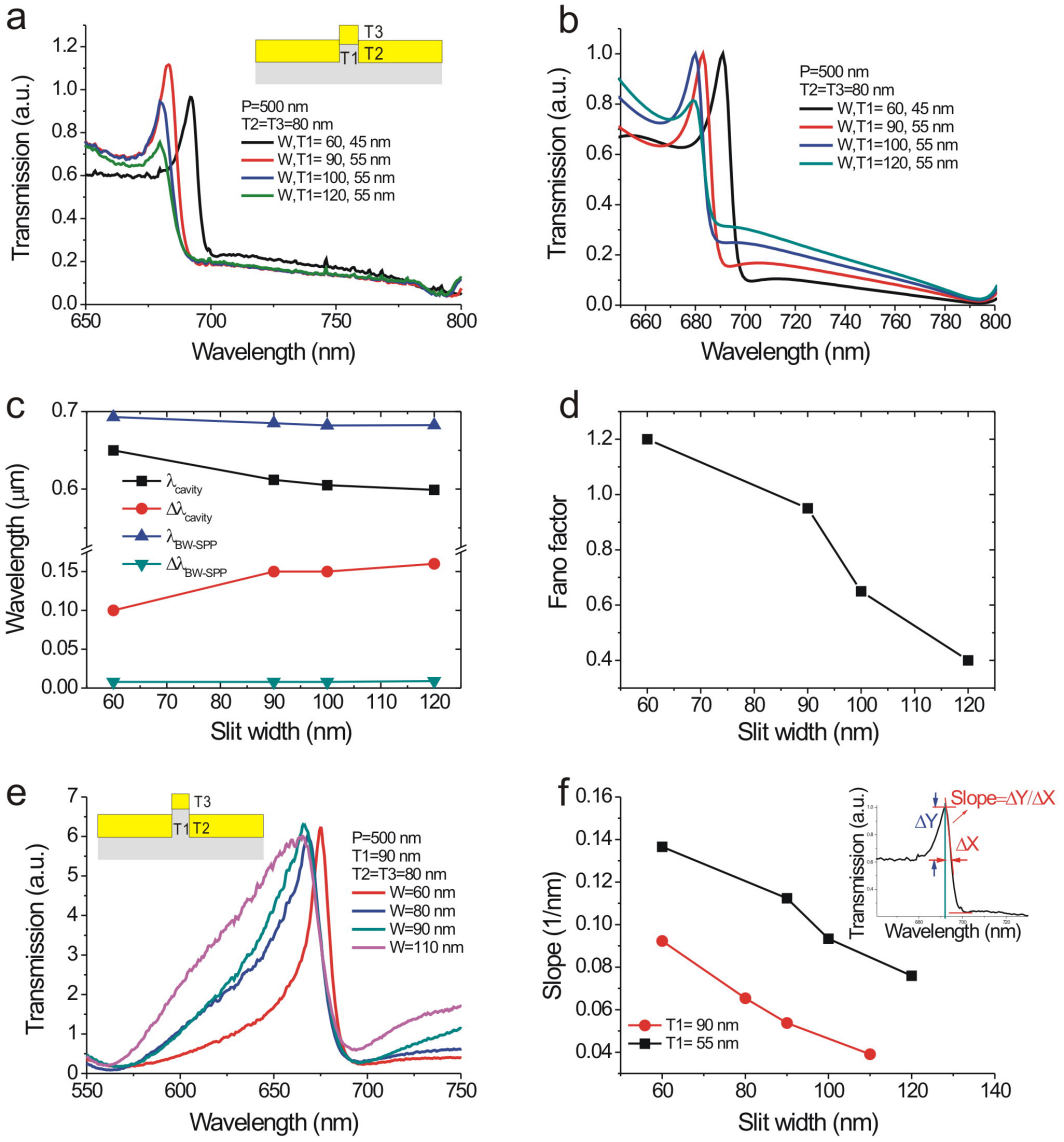
The effect of the slit width on the transmission spectra of long- and short-cavity capped nanoslits with a period of 500 nm in water for normally-incident TM-polarized light. (a) The transmission spectra of the short-cavity capped gold nanoslits. (b) The calculated Fano resonance spectra for different slit widths using equation (2). (c) The fitting resonant wavelengths and bandwidths of the cavity mode and BW-SPP mode as a function of the slit width. (d) The Fano asymmetrical factor as a function of the slit width. (e) The transmission spectra of the long-cavity capped gold nanoslits for different slit widths. (f) The resonant slope as a function of the slit width for both short- and long-cavity capped structures. The inset depicts the definition of the resonant slope.

In addition to the cavity length, the slit width plays an important role to the Fano resonance. We compared the spectra of the capped gold nanoslit arrays with various slit widths in water. The structure parameters were P = 500 nm, T2 = T3 = 80 nm, T1 = 45–90 nm and W = 60–120 nm. The transmission spectra of short cavity length (T1 = 45, 55 nm) with various slit widths are shown in Figure 3a. A sharper resonant profile was formed for a smaller slit width. Figure 3b shows the fitting spectra using equation (2). Figure 3c shows the fitting parameters as a function of the slit width. The resonant wavelengths of cavity modes and BW-SPP modes were redshifted with a decrease of the slit width. It is noted that there is a substantially decrease of the cavity bandwidth as the slit width decreases. In the nanoslits, there is no cut-off for the TM polarized wave. The TM wave can propagate along the slit gaps. Due to the reflection at upper and lower interfaces, a Fabry-Perot cavity is formed^33^. The propagation constant inside the slit, the phase of reflection, and the amplitude of reflection can influence the transmission properties through the slit. For the cavity mode, the optical wave is in resonance when it satisfies
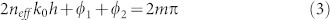
where n_eff_ is the equivalent refractive index in the slit, k_0_ is the free space wavelength vector (2π/λ_0_), h is the cavity length (T2), and ϕ_i_ is the phase of reflection at the upper/lower interface of the gold slit. As the slit becomes narrower, there is more penetration of the field into the metal so that both the n_eff_ and attenuation coefficient increase. The n_eff_ increases with the decrease of the slit width^33^,^36^. As a result, the resonant wavelength of the cavity mode increases when the slit width is reduced. On the other hand, the quality of the Fabry-Perot resonance is related to the amplitude of reflection at the upper/lower interface of the gold slit^33^. The increased effective index in the slit results in a higher effective-index contrast and tighter vertical confinement of the mode and thus increases the amplitude of reflection. Therefore, a narrower nanoslit has a sharper cavity mode^36^,^37^. The propagation loss is not the dominating loss mechanism in the cavity because the cavity length, h, is very short. Figure 3d shows the Fano factors as a function of the slit width. The factor was increased with the decrease of the slit width. The short-cavity capped nanoslit with a slit width of 60 nm had a higher Fano coupling efficiency and sharper resonant peak. Figure 3e shows the measured spectra with long-cavity capped nanoslits (T1 = 90 nm) and different slit widths. The spectra also show increased sharpness when the slit width was reduced. We further compared the sharpness of the resonant peaks for long-cavity and short-cavity capped nanoslit arrays. Figure 3f shows the results. The definition of the resonant slope was shown in the inset. Obviously, the slope was increased with the reduction of the slit width. Compared to the long-cavity capped nanoslits, the short-cavity one had a higher slope and narrower bandwidth.

### Refractive index sensing capabilities of the capped gold nanoslits

**Figure 4:**
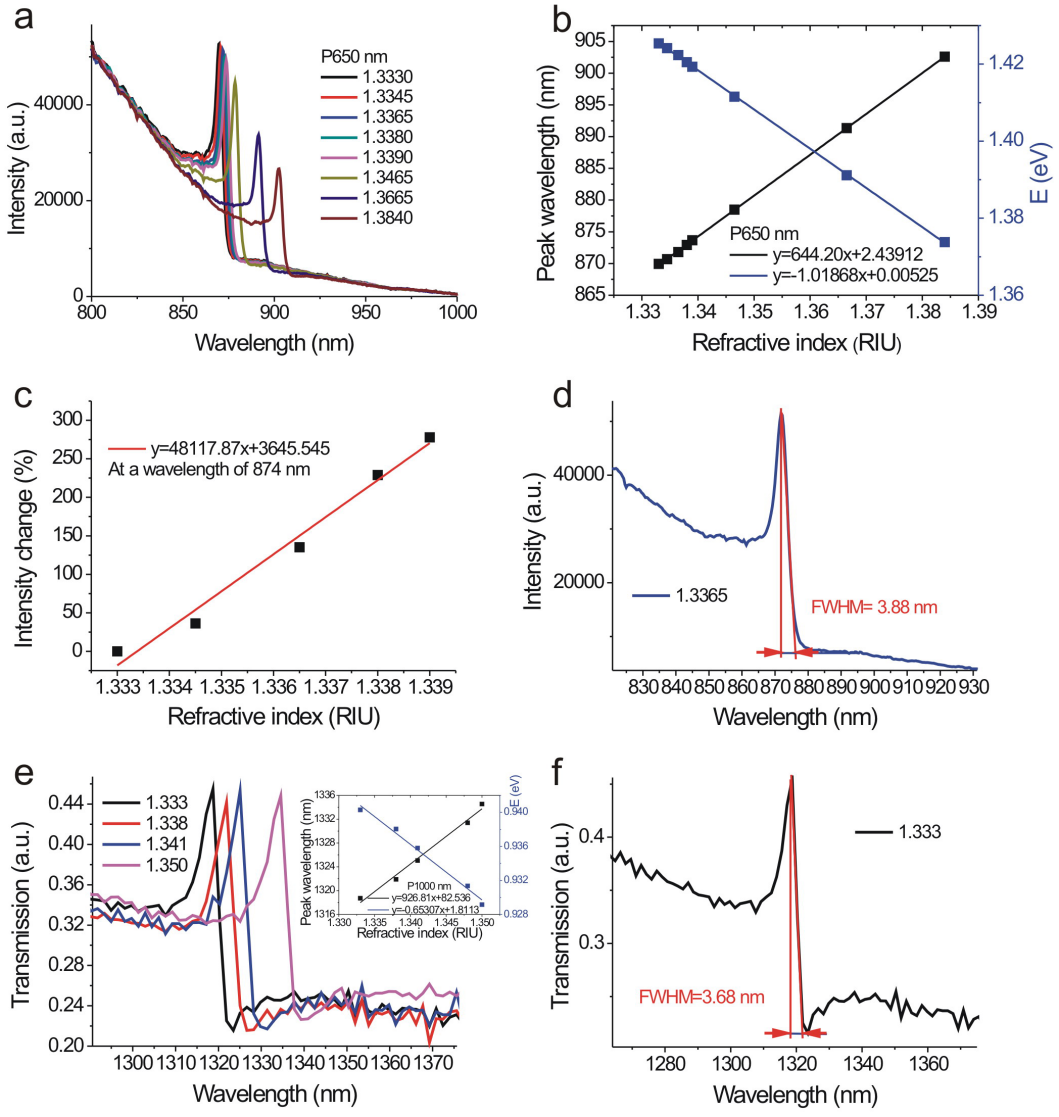
Refractive index sensing capabilities of the capped gold nanoslits made by the template stripping method. (a) The intensity spectra of the capped gold nanoslits with a 650-nm period in various water/glycerin mixtures for a normally-incident TM-polarized wave. (b) The resonant peak wavelength and energy against the refractive index of the outside medium. The slopes of the fitting curves show that the refractive index sensitivities were 644 nm/RIU and 1.018 eV/RIU. (c) The normalized intensity change against the refractive index of the outside medium. The slope of the fitting curve shows that the intensity sensitivity was 48,117%/RIU. (d) The measured fwhm bandwidth of the Fano resonant peak in glycerin/water mixture (n = 1.3365) was 3.88 (fwhm = 0.0063 eV). The extremely sharp resonance leads to a high-quality factor up to 224. (e) The transmission spectrum of the capped nanoslits with a period of 1,000 nm in various water/glycerin mixtures for normally-incident TM-polarized light. The inset shows the refractive index sensitivities were 926 nm/RIU and 0.653 eV/RIU. (f) The enlarged transmission spectrum of the capped nanoslits with a period of 1,000 nm in water. The measured fwhm bandwidth of the Fano resonant peak was 3.68 nm (0.0026 eV).

To verify the high sensitivity of the capped gold nanoslits, we tested the refractive index sensitivity of the nanostructure with a period of 650 nm. The bulk sensitivity of the sensor was measured by injecting purified water mixed with various ratios of glycerin into the microfluidic devices. Figure 4a shows the intensity spectra of the capped gold nanoslits with various water/glycerin mixtures for a normally-incident TM-polarized wave. There were sharp Fano resonances in the spectra. The wavelength of the Fano resonance at the water/gold interface was near 870 nm. When the concentrations of glycerin increased, the wavelength of Fano resonance was redshifted and the intensity changed. Figures 4b shows the resonant peak wavelength and intensity against the refractive index of the outside medium. The slopes of the fitting curves show that the refractive index sensitivities were 644 nm/RIU and 1.018 eV/RIU. Figure 4c shows the normalized intensity change against the refractive index of the outside medium. The slope of the fitting curve shows that the intensity sensitivity was 48,117%/RIU. The measured intensity sensitivity is much better than the reported intensity sensitivities of nanoslit, nanohole or nanogrid arrays ~1,010–12,963%/RIU^12^,^21^,^38^ and prism-based SPR sensors ~15,000%/RIU^2^. For the current system, the integration time for acquiring one spectrum was 125 milliseconds and the intensity noise was 1.8%. Thus, the detectable refractive index resolution was 3.74 × 10^−5^ RIU. If the intensity stability is further reduced to 0.2%, the structure can achieve a bulk refractive index resolution of 4.15 × 10^−6^ RIU. Such a sensitivity is comparable with commercial SPR machines using complicated high-resolution angular detection method. To compare the refractive index sensing capability of the fabricated nanostructures with previous works, we also calculated the figure of merit (FOM) values in wavelength units and energy units. The FOM in energy units (FOM_E_) is defined as m (eV/RIU)/fwhm (eV), where m is the linear regression slope for the refractive index dependence and fwhm is the resonance width of the plasmon resonance^39^. In Figure 4d and 4b, the measured fwhm bandwidth of the Fano resonant peak in glycerin/water mixture (n = 1.3365) was 3.88 nm (fwhm = 0.0063 eV) and the wavelength sensitivity was 644 nm/RIU (1.018 eV/RIU). Thus, the FOM (FOM_E_) value of 166 (162) was obtained. The quality factor (resonant wavelength/line width) of the system was 224 (870 nm/3.88 nm). The obtained value (FOM = 166) is higher than the theoretically estimated upper limits (FOM = 108) of the prism-coupling SPR sensors^40^, the previously reported record high FOM in nanohole sensors^26^,^29^ and the LSPR sensors^9^,^27^,^28^. For example, the periodic gold nanohole arrays (FOM of 23 and 162)^26^,^29^, plasmonic gold mushroom arrays (FOM of 108)^30^, single slit with grooves (FOM of 48)^31^, various shapes of gold nanoparticles (FOM_E_ = 0.6–4.5)^9^ and the reported plasmonic nanostructures and metamaterials with Fano-type resonances (a non-concentric ring/disk cavity (FOM of 8.34)^27^, plasmonic nanoparticle clusters (FOM of 10.6)^28^ and an electromagnetically induced transparency (EIT) based planar metamaterial (FOM of 5.3))^32^. It is noted that the FOM value can be further improved when the period of nanostructure is increased. Figure 4e shows the normalized transmission spectrum of the capped nanoslits with a period of 1,000 nm in various water/glycerin mixtures for normally-incident TM-polarized light. The measured fwhm bandwidth of the Fano resonant peak was 3.68 nm (0.0026 eV) (See Figure 4f). As the wavelength sensitivity is proportional to the period, the wavelength sensitivity was further increased to 926 nm/RIU (0.653 eV/RIU). Thus, the FOM value was up to 252 (FOM_E_ = 251). In addition, the quality factor (resonant wavelength/line width) of the system was 358 (1,318 nm/3.68 nm).

### Biosensing using the Fano resonance in 600-nm-period capped gold nanoslits

**Figure 5:**
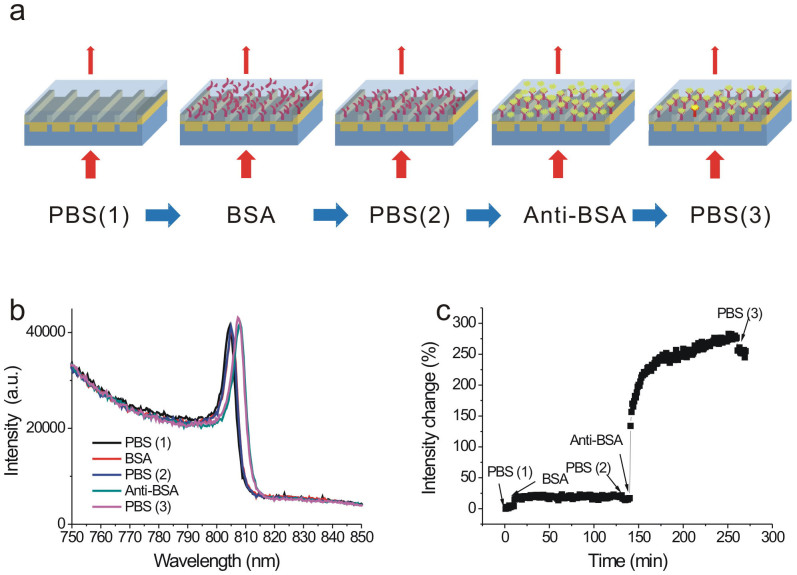
Biosensing using the Fano resonance in 600-nm-period capped gold nanoslits. (a) A process flowchart for the interactions between BSA and anti-BSA. (b) The measured intensity spectra for different surface conditions. Significant changes in wavelength shift were observed when BSA and anti-BSA were bound on the gold surface. (c) The normalized intensity change of as a function of the interaction time for protein-protein interactions. The BSA resulted in an intensity change of 13%. The Anti-BSA caused an intensity change of 237%.

We further conducted a protein-protein interaction experiment to verify the high intensity sensitivity. The interactions between bovine serum albumin (BSA) and anti-BSA were measured using capped gold nanoslits with a 600 nm period, as shown in Figure 5a. First, the buffer solution, 10 mM phosphate-buffered saline (PBS), was first injected to the fluidic channel to clean the gold surface. The time-lapsed intensity spectra of the nanostructure in buffer solution were recorded with a measuring period of 1 min. Then 500 μg/mL BSA was injected on the structure surface. Due to the physical adsorption of BSA on the gold surface, a BSA monolayer coated on the surface. After two hours of static adsorption, sufficient BSA immobilized on the gold surface. Next, the sample was washed by PBS buffer to remove the unbound BSA proteins. Afterward, 375 μg/mL anti-BSA was injected into the sample. Finally, the unbound anti-BSA was washed away by the PBS buffer after two hours of interaction. Figure 5b shows the measured spectra for different surface conditions. Significant changes in wavelength shift were observed when BSA and anti-BSA were bound on the gold surface. The monolayer BSA resulted in a 0.40-nm red shift. The wavelength shift is small because BSA is a small molecule with 66 kDa in size. The wavelength shift for anti-BSA molecules was large. The 150-kDa-sized anti-BSA resulted in a 2.76-nm wavelength shift. If the wavelength resolution is 0.02 nm, the signal-to-noise ratio is 138 for the anti-BSA. One the other hand, the sharp slope of the Fano resonance resulted in an ultrahigh intensity sensitivity. Figure 5c shows the intensity change of as a function of the interaction time for protein-protein interactions. The measured intensity signal is at a wavelength of 810 nm. It is stable with time when the PBS buffer is injected into the microfluidic device. The BSA coated on the gold surface resulted in an intensity change of 13%. For the Anti-BSA, the 375 μg/mL concentration caused an intensity change up to 237%. Using the intensity measurement, the signal-to-noise ratio for Anti-BSA molecules is up to 1185 when the intensity stability is 0.2%. Therefore, the capped nanoslit array has a higher signal-to-noise ratio for the intensity measurement than that for the wavelength measurement.

## Discussion

We fabricated large-area capped gold nanoslits on a polycarbonate substrate for intensity-sensitive detection using a thermal annealing-assisted template stripping method. The experimental results show that a transverse magnetic-polarized wave in the gold nanostructures generated extremely sharp and asymmetric Fano resonances in transmission spectra. The full width at half-maximum bandwidth is 3.68 nm (0.0026 eV) and the wavelength sensitivity is 926 nm/RIU (0.653 eV/RIU) for 1,000-nm-period capped gold nanoslits. The extremely sharp resonance leads to a high-quality factor up to 358. Compared to the long-cavity capped nanoslits and uncapped nanoslits, the short-cavity capped nanoslits has a similar wavelength sensitivity but a narrower bandwidth. It reaches a record high figure of merit (FOM) up to 252 (FOM_E_ = 251). In addition, the short-cavity structure has a higher intensity sensitivity up to 48,117%/RIU. It is noted that plasmonic sensing is not suited for molecular identification. However, the proposed structures can be utilized to combine plasmonic sensing with surface enhanced Raman scattering (SERS) detection techniques^41^ so that the probe molecules can be characterized and then identified to increase the reliability of biological detection. In addition, the proposed structure with highly sensitive Fano resonances can be applied to develop colorimetric test strips for point-of-care applications. Such inexpensive, reproducible and high-throughput fabrication of highly sensitive capped nanoslits can benefit sensing applications.

## Methods

### Fabrication of silicon template

**Figure 6:**
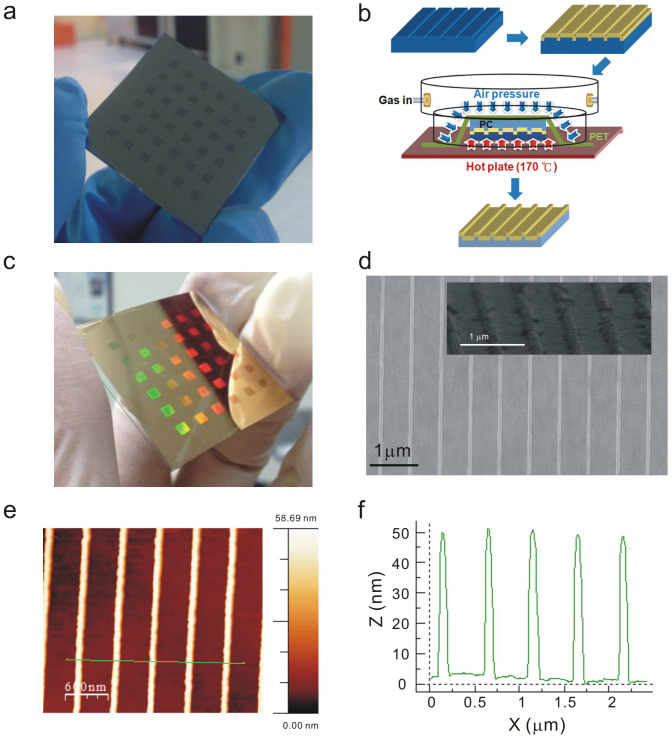
Fabrication of capped gold nanoslits using a thermal annealing-assisted template stripping method. (a) The optical image of the fabricated silicon template. There are 6 × 6 groove arrays on the silicon stamp. The area of each array is 2 × 2 mm^2^. The groove width, depth and period are 60, 50 and 500 nm, respectively. (b) A process flowchart for the fabrication of metallic nanostructures. (c) The optical image of the template-stripped nanostructures on a plastic film. (d) The SEM image of the template-stripped nanostructures. The inset shows the enlarged SEM image with a viewing angle of 45 degrees. (e) The atomic force microscopy images of the template-stripped nanostructures. (f) A cross-sectional profile of template-stripped nanostructures. The height of the capped gold film (T1) is about 50 nm.

Nanoslit arrays on a silicon substrate were fabricated using electron beam lithography and a reactive ion etching method. A 300-nm-thick ZEP-520 resist (ZEP-520, Zeon Corp, Tokyo, Japan) was spin-coated on a 525-μm-thick silicon substrate. An electron-beam drawing system (Elionix ELS 7,000) was used to write nanoslit arrays with various slit widths. The patterns were then transferred to the silicon substrate by using a reactive ion etching machine (Oxford Instrument, plasmalab 80plus). The power of the radiofrequency (RF) electromagnetic wave in the reaction chamber was 150 W. The chamber pressure was 1 × 10^−2^ torr and the flow rates of CHF_3_ and SF_6_ gases were 50 sccm and 25 sccm, respectively. The resist was removed by rinsing the sample in acetone for a few minutes, before it was put in ultrapure water and purged dry by nitrogen. In the experiment, nanoslit arrays with various periods, slit depths and slit widths were made. The period varied from 500 to 1,000 nm, the depth from 45 to 180 nm and the width from 50 to 140 nm. Figure 6a shows the optical image of the fabricated silicon template. There are 6 × 6 nanoslit arrays on the silicon stamp. The area of each array is 2 × 2 mm^2^. The groove width, depth and period are 60, 50 and 500 nm, respectively.

### Fabrication of metallic nanostructures

Metallic nanostructures were made on a polycarbonate (PC) substrate using a thermal annealing-assisted template stripping method^21^. Figure 6b depicts a process flowchart for the fabrication of metallic nanostructures. An 80-nm-thick gold film was deposited at a slow deposition rate (0.1 nm/sec) on the clean silicon template using an electron gun evaporator. A 178-μm-thick PC film (Lexan8010, GE, USA) was placed on the gold coated template. The template and PC substrate was placed on a heating plate. It was heated at a temperature of 170°C to soften the PC substrate. An additional polyethylene terephthalate (PET) thin film was used as the sealing film. In the system, nitrogen gas was introduced into the chamber to produce a uniform pressure (2 kgw/cm^2^) over the film. It pressed the silicon mold and PC substrate with large-area uniformity. This step made the gold film uniformly stuck to the softened PC film. The template and substrate were then cooled and taken out from the chamber. As the gold film had a poor adhesion to the silicon template, the PC film was easily separated from the silicon template. After peeling off from the template and PET thin film, the PC substrate with metallic nanostructures was made. It was noted that the slit depths of the silicon template for fabricating short-cavity, long-cavity capped nanoslits and uncapped nanoslits, were 55, 90 and 180 nm, respectively. The high aspect ratio of the nanoslit limited the depth of the penetration of the softened PC film. It prevented the gold strips in the slits from sticking to the softened PC film. Only the gold film on the template stuck to the softened PC film. Therefore, uncapped nanoslits were made using the template with high-aspect-ratio grooves. On the other hand, the gold strips in the low-aspect-ratio nanoslits can easily stick to the softened PC film and the capped nanoslits were made. Figure 6c shows the optical image of the template-stripped nanostructures on a plastic film. The scanning electron microscope (SEM) image of the template-stripped nanostructures is shown in Figure 6d. The inset shows the enlarged SEM image with a viewing angle of 45 degrees.

### Optical setup for transmission spectrum measurement

The transmission spectra were measured by a simple optical transmission setup^14^. A 12W halogen light was spatially filtered by using an iris diaphragm and a collimation lens. Its incident polarization was controlled by a linear polarizer. The white light was focused on a single array by using a 10× objective lens. The transmission light was collected by another 10× objective lens and focused on a fiber cable. The transmission spectrum was taken by using a fiber-coupled linear CCD array spectrometer (BWTEK, BTC112E and BTB 1,100). In the experiment, the metallic nanostructures were mounted on a microfluidic channel made from plexiglass. The refractive index sensitivities were measured by covering purified water mixed with various fractions of glycerin over the sample surface. The refractive indexes of the mixtures (from 0 to 15% glycerin) were measured with a refractometer and ranged from 1.333 to 1.384. The biosensing experiments were conducted by using BSA (Sigma-Aldrich) and anti-BSA (Sigma-Aldrich) assay in 10 mM PBS (UniRegion Bio-Tech) buffer condition.

### Atomic force microscopy (AFM) measurements

All atomic force microscopy images were obtained with a Veeco di Innova AFM instrument operating in tapping mode in air. The scan size of the AFM image is typically 5 × 5 μm^2^ at a scan rate of 0.8 Hz. Figure 6e shows the atomic force microscopy images of the template-stripped nanostructures. A cross-sectional profile of template-stripped nanostructures is shown in Figure 6f. The depth of grooves on the upper side is about 50 nm.

## Author Contributions

P.K.W. and K.L.L. conceived and designed the experiments; K.L.L., J.B.H., J.W.C. and S.H.W. performed the experiments; K.L.L. and P.K.W. analyzed the data; P.K.W. contributed materials/analysis tools; K.L.L. and P.K.W. co-wrote the paper.
